# Ultrasound versus anatomical landmarks for caudal epidural anesthesia in pediatric patients

**DOI:** 10.1186/s12871-015-0082-0

**Published:** 2015-07-14

**Authors:** Yukako Abukawa, Koichi Hiroki, Nobutada Morioka, Hiroko Iwakiri, Tomoko Fukada, Hideyuki Higuchi, Makoto Ozaki

**Affiliations:** 1 Department of Anesthesia and Critical Care, Tokyo Women’s Medical University, Tokyo, Japan; 2 Kanagawa Children’s Medical Center, 4-1124-2 Mutsukawa, Minami-ku, Yokohama, Kanagawa 232-0066 Japan

**Keywords:** Caudal epidural anesthesia, Ultrasound, Anatomical landmark

## Abstract

**Background:**

Caudal block is easily performed because the landmarks are superficial. However, the sacral hiatus is small and shallow in pediatric patients. In the present study, we evaluated under general anesthesia whether the distance between the bilateral superolateral sacral crests increased with growth, whether an equilateral triangle was formed between the apex of the sacral hiatus and the bilateral superolateral sacral crests, and whether expansion of the epidural space could be confirmed by ultrasound.

**Methods:**

This prospective observational study included 282 children who were ASA I–II. Under general anesthesia, each patient was placed in the lateral bent knees position, and the attending anesthesiologist drew an equilateral triangle and measured the distance between the bilateral superolateral sacral crests along a line forming the base of the triangle. Then the sacral hiatus was identified by ultrasound. Differences of the distance between the anatomical landmarks measured by the anesthetist and by ultrasound were evaluated.

**Results:**

Two patients were excluded because the superolateral sacral crests and sacral hiatus could not be palpated. The base of the triangle increased in proportion to age up to 10 years old, with a significant correlation between age and the length of the base (Spearman’s *r* value = 0.97). The triangle was not an equilateral triangle under 7 years old. The sacral hiatus could be identified by ultrasound and we could confirm expansion of the epidural space in all patients.

**Conclusion:**

We observed a correlation between age and the length of the triangle base in children under 10 years old. Although detection of the anatomical landmarks by palpation differed from identification by ultrasound in pediatric patients, performing ultrasound is important. Epinephrine should be added to the anesthetic to avoid complications.

**Trial registration:**

Current Controlled Trials UMIN000017898. Registered 14 June 2015. Date of protocol fixation was 1^st^ December, 2008 and Anticipated trial start date was 5^th^ January, 2009.

## Background

Caudal epidural block can be performed easily because the landmarks are superficial. However, the pediatric sacral hiatus is small and shallow. In infants, the needle should not be advanced more than 2 mm after loss of resistance is noted because the dural sac and epidural veins terminate at S_3–4_. Also, there are many variations of the sacral hiatus and sacral cornua [1]. In fact, it has been reported that the success rate of caudal epidural analgesia is only 75 % in pediatric patients [2, 3]. In adults, the triangle between the apex of the sacral hiatus and the bilateral superolateral sacral crests is approximately an equilateral triangle [4], but the relation among these landmarks has not been assessed in pediatric patients. Therefore, in pediatric patients under general anesthesia, we evaluated whether the distance between the bilateral superolateral sacral crests increased with growth, whether an equilateral triangle was formed by the apex of the sacral hiatus and the bilateral superolateral sacral crests, and whether expansion of the epidural space could be confirmed by ultrasound.

## Methods

This study was approved by ethics committee in Intelligent Clinical Research and Innovation Center at Tokyo Women’s Medical University, and oral and written informed consent was obtained from the parents of all patients. The study population was derived from the patients who received caudal epidural anesthesia from anesthetists of the Department of Anesthesia at Tokyo Women’s Medical University Hospital between September 1, 2009 and January 1, 2014. This prospective observational study included 282 children aged from one month to 10 years, with an ASA physical status of I–II (Table 1). Under general anesthesia, each patient was placed in the lateral bent knees position, and the attending anesthesiologist drew the anatomical landmarks on the patient’s body. The anesthesiologist also drew an equilateral triangle and measured the distance along a line between the two superolateral sacral crests, which formed the base of the triangle. Then the sacral hiatus was identified by ultrasound before puncturing the skin. (It should be noted that ultrasound-guided anesthesia was not performed). Next, a 25 gauge B-bevel needle (Unisys Corp., Japan) was inserted until a marked decrease of resistance was noted, followed by the injection of 1 ml of levobupivacaine containing 1:200,000 epinephrine. The heart rate, blood pressure, and ST changes were monitored throughout. A test dose was injected first and expansion of the epidural space was evaluated by ultrasound, and any effects on the heart rate and T wave were also monitored. If the heart rate increased by ≥10 %, the needle was removed and then re-inserted. Caudal epidural block was achieved by injection of 0.25 % levobupivacaine containing 1:200000 epinephrine (1 ml/kg). Differences between identification of the anatomical landmarks by palpation and by ultrasound were evaluated.

**Table 1:** Patient characteristics

Total number	Gender (M/F)	Age (Mo)	Weight (kg)	Height (cm)
*N* = 280	241/39	36 ± 25	13 ± 5	89 ± 17

Data are expressed as the mean ± standard deviation (SD)

The base of the triangle was measured between the anatomical landmarks and by ultrasound and the difference was compared before puncturing the skin. We evaluated whether an equilateral triangle was formed by the apex of the sacral hiatus and the bilateral superolateral sacral crests, whether the distance between the two superolateral sacral crests was correlated with growth, and whether expansion of the epidural space could be evaluated by ultrasound.

The relationship between age and the length of the base of the triangle was determined by Spearman’s rank correlation analysis. Results are expressed as the mean ± standard deviation (SD). Statistical analyses were performed using the Graph Pad Prism 5 software package.

## Results

**Fig. 1:**
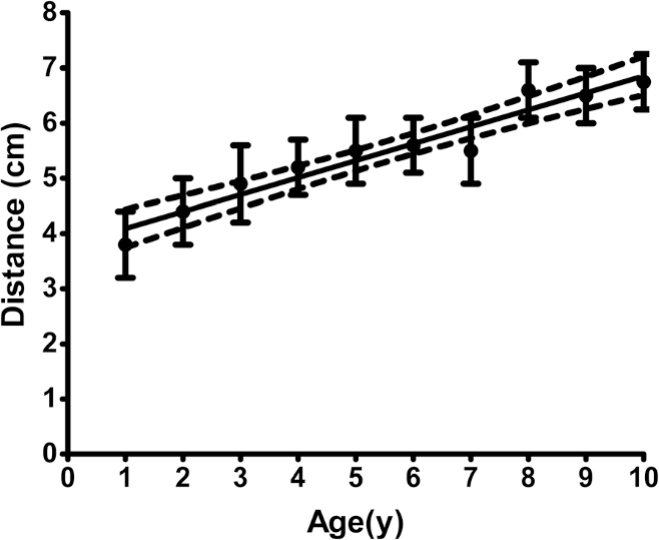
Correlation between age and the length of the triangle base in children under 10 years old. Age is plotted versus the distance between the bilateral superolateral sacral crests. There was a significant positive correlation between age and the distance between the two crests up to the age of 10 years. Spearman’s *r* value was 0.97

Two patients were excluded because the anatomical landmarks could not be palpated. The characteristics of the remaining 280 patients are shown in Table 1. The mean duration of anesthesia was 140 ± 65 min and the mean operating time was 88 ± 60 min. None of the patients had any complications and postoperative pain was easily controlled in all of them. The mean distance between the right and left superolateral sacral crests (the base of the triangle) was 4.9 ± 0.9 cm (Table 2). There was a significant positive correlation between age and the length of the triangle base up to the age of 10 years (Spearman’s *r* value = 0.97; *p* < 0.0001) (Fig. 1).

**Table 2:** Anatomical measurements

Distance between the two superolateral sacral crests (base of the triangle; cm)	
1 mo ~ 10y	4.9 ± 0.9
1 mo ~ 1 y	3.8 ± 0.6
1 ~ 2 y	4.4 ± 0.6
2 ~ 3 y	4.9 ± 0.6
3 ~ 4 y	5.2 ± 0.5
4 ~ 5 y	5.5 ± 0.6
5 ~ 6 y	5.6 ± 0.5
6 ~ 7 y	5.5 ± 0.6
7 ~ 8 y	6.6 ± 0.5
8 ~ 9 y	6.5 ± 0.5
9 ~ 10 y	6.8 ± 0.5
Difference between anatomical landmarks and ultrasound (cm)	
1mo ~ 10y	0.7 ± 0.5
Percent difference (%)	
1 mo ~ 10y	14 ± 10
1 mo ~ 1 y	13.6 ± 9.9
1 ~ 2 y	16.0 ± 9.9
2 ~ 3 y	15.1 ± 10.0
3 ~ 4 y	14.3 ± 9.1
4 ~ 5 y	15.6 ± 12.2
5 ~ 6 y	13.9 ± 8.8
6 ~ 7 y	12.6 ± 9.3
7 ~ 8 y	4.0 ± 6.8
8 ~ 9 y	6.0 ± 10.4
9 ~ 10 y	0.7 ± 1.6

Data are expressed as the mean ± standard deviation (SD)

**Fig. 2:**
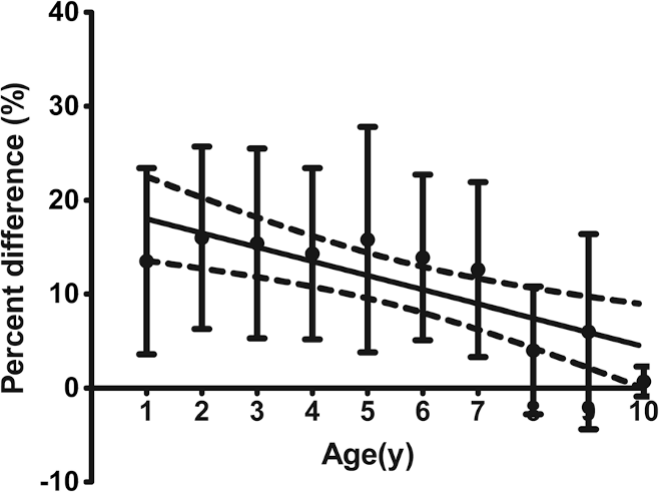
Correlation between age and the percent difference. The difference of the distance between the two superolateral sacral crests (anatomical landmarks) and identification by ultrasound was measured. The horizontal axis shows age and the vertical axis shows the percent difference between the anatomical landmarks and identification by ultrasound

It was difficult to detect the sacral hiatus by palpation in two patients (0.7 %) because of anatomical abnormality and obesity. However, ultrasound successfully identified the sacral hiatus in all patients. The heart rate increased after injection of the test dose in one patient (0.4 %) even though expansion of the epidural space was seen on ultrasound. Therefore, the needle was reinserted into the epidural space at another site and intravenous injection could be detected using epinephrine. There was no correlation between the percent difference and age (Fig. 2).

## Discussion

To our knowledge, this is the first report on initial anatomical measurements for caudal epidural anesthesia in children aged from one month to 10 years. We found that the distance between the right and left superolateral sacral crests increased in proportion with age up to 10 years old. However, there was a difference between identification of the anatomical landmarks by palpation and by ultrasound, and it was found that the isosceles triangle formed by the landmarks became closer to an equilateral triangle as the children got older. These results are useful for identifying the sacral hiatus in children. It is helpful to avoid the need for local anesthesia by using a short needle for caudal block. Most of the patients in this study were boys because the operations were orchiopexy (60 %), circumcision (5 %), inguinal hernia repair (20 %), and repair of hypospadias (15 %).

There were three important findings of this study. The first is the importance of determining the depth of the epidural space below the skin to avoid dural puncture. Ultrasound is effective for determining the depth of the epidural space and for observing expansion of the epidural space during injection of local anesthetic. However, latent vascular injection of local anesthetic is not detected by ultrasound. An excess dose of local anesthetic (1 ml/kg) is usually employed for pediatric caudal anesthesia. We used 0.25 % levobupivacaine (1 ml/kg) for caudal analgesia [5], which was combined with 1:200000 epinephrine to avoid vascular injection and intoxication. According to Adewale et al., there was no correlation between the maximum depth of the epidural space measured by magnetic resonance imaging and the age, height, weight, or body surface area in children [6]. Therefore, it is valuable to be able to determine the depth of caudal space by ultrasound. Our second important finding was that there was a difference between identifying the anatomical landmarks by palpation and by ultrasound, and that the isosceles triangle formed by the landmarks became closer to an equilateral triangle as the children got older This result is useful for identifying the sacral hiatus in children. The third finding was that the distance between the bilateral superolateral sacral crests increased with age and there was a correlation between this distance (the base of the triangle) and age up to 10 years old. The main limitation of this study was the small number of children over 7 years old, so more observational studies are needed to increase the data on older patients.

There are several variations in the location and presence of the sacral hiatus in adults [1]. About 4 % of patients do not have a sacral hiatus and 53 % do not have a sacral cornua. Such variations of the landmarks can make it difficult to perform caudal epidural anesthesia in pediatric patients, and the failure rate of caudal anesthesia in children is 25 % [2, 3]. Examination of the caudal space in fetuses showed that the anatomical landmarks seem to form an isosceles triangle, with the two superolateral sacral crests at the base and do not form an equilateral triangle [7, 8].

The prevalence of occult spinal dysraphism (OSD) is higher in children under 24 months old with simple urogenital anomalies than in the general population [9]. Ultrasound is minimally invasive and can be performed easily and rapidly, especially pediatric patients.

## Conclusions

In summary, we observed a correlation between age and the length of the triangle base in children under 10 years old. Identification of the sacral hiatus by anatomical landmarks and ultrasound differed somewhat in pediatric patients, so it is important to check patients by ultrasound and to add epinephrine to the anesthetic to avoid complications. Based on the report of Koo et al., it is especially important to assess the spinal structures prior to performing caudal bock in children under 2 years old with urological anomalies.
